# Identifying and addressing underuse in hematologic care through systems-based hematology

**DOI:** 10.1016/j.rpth.2025.102881

**Published:** 2025-05-08

**Authors:** Jacob C. Cogan, Allison E. Burnett, Alexandra Power-Hays, Geoffrey D. Barnes, Ming Y. Lim

**Affiliations:** 1 Division of Hematology, Oncology and Transplantation, University of Minnesota, Minneapolis, Minnesota, USA; 2 College of Pharmacy, University of New Mexico Hospital, Albuquerque, New Mexico, USA; 3 Division of Hematology, Cincinnati Children’s Hospital Medical Center, Cincinnati, Ohio, USA; 4 Department of Pediatrics, University of Cincinnati, Cincinnati, Ohio, USA; 5 Division of Cardiovascular Medicine, Department of Internal Medicine, Frankel Cardiovascular Center, University of Michigan, Ann Arbor, Michigan, USA; 6 Division of Hematology and Hematologic Malignancies, Department of Internal Medicine, University of Utah, Salt Lake City, Utah, USA

**Keywords:** anticoagulation, hydroxyurea, IVC filter, sickle cell anemia, underuse

## Abstract

Underuse of high-value hematologic care receives comparatively less attention than the overuse of unnecessary tests and treatments. In this review, we analyze examples of underutilized care in several domains: procedural (inferior vena cava filter retrieval), medical (hydroxyurea use in sickle cell anemia), and patient-facing (education prior to anticoagulation). For each, we justify the need for increased uptake and highlight examples of systems-based hematology interventions to accomplish this. While reducing overused care is appealing from a cost savings perspective, we advocate for equal attention and investment toward promoting underused care.

## Essentials

• Underuse of high-value hematologic care receives less attention than overuse.
• Inferior vena cava retrieval is increased through active and passive surveillance programs.
• Hydroxyurea use in sickle cell anemia expands with technological and multidisciplinary efforts.
• System-level patient education processes may improve rates of anticoagulation patient education.

## 1 Introduction

As health systems increasingly prioritize the provision of high-value care, the overuse and underuse of medical services have emerged as important barriers. These improper use patterns plague healthcare delivery, from publicly funded to private health systems and fee-for-service to salaried providers [1]. The drivers of overuse and underuse are complex and substantial, involving economic incentives and human psychology [2], and concerted, combative efforts are required to address them.

Overuse of hematologic care has received more attention than underuse. As part of the American Board of Internal Medicine Choosing Wisely campaign, the American Society of Hematology developed a list of recommendations focused on reducing the overuse of services such as blood transfusions, thrombophilia testing, and imaging studies (Table) [[3], [4], [5], [6], [7], [8]]. These recommendations have spurred efforts at institutions throughout the country to promote their adoption [9]. While reducing overused care is appealing from a cost savings perspective, there is room for equal attention and investment toward increasing effective but underutilized care.

**Table:** Examples of overused and underused interventions in hematology.

Overused interventions	Underused interventions
IVC filter placement in acute VTE [3,4]^a^	IVC filter retrieval^b^
Thrombophilia testing patients with provoked VTE [3,4]^a^	Hydroxyurea use in SCA^b^
Plasma or PCC for nonemergent reversal of vitamin K antagonists [3,4]^a^	Patient education prior to anticoagulation^b^
Plasma or PCC for elevated INR in patients with cirrhosis	Use of the 4T score to guide diagnostic evaluation for heparin induced thrombocytopenia [5,6]
Surveillance CT scans in asymptomatic patients after curative-intent lymphoma treatment [3,4]^a^	Treatment of iron deficiency in patients without anemia [7,8]
Transfusion of multiple red blood cell units to return hemoglobin to a safe range (eg, >7 g/dL) [3,4]^a^	Clinical trial enrollment in patients with classical hematologic diseases

CT, computed tomography; INR, international normalized ratio; IVC, inferior vena cava; PCC, prothrombin complex concentrates; SCA, sickle cell anemia; VTE, venous thromboembolism.

a Present in the American Society of Hematology Choosing Wisely list.

b Covered in this article.

Underuse in healthcare refers to the failure to deliver a service that is likely to improve the quality or quantity of life, has favorable cost-benefit tradeoffs, and would be desired by patients informed about the risks and benefits [10]. As institutions invest in hiring systems-based hematologists—providers focused on developing pathways to deliver high-quality, cost-effective, and evidence-based care to patients with blood disorders across healthcare systems [11,12]—the present is an opportune time to promote underused hematologic care. In this review, we describe examples of systems-based hematology interventions to promote several forms of care: removal of inferior vena cava (IVC) filters, increased use of hydroxyurea in sickle cell anemia (SCA), and provision of education prior to anticoagulation treatment.

## 2 IVC Filter Retrieval

**Figure 1:**
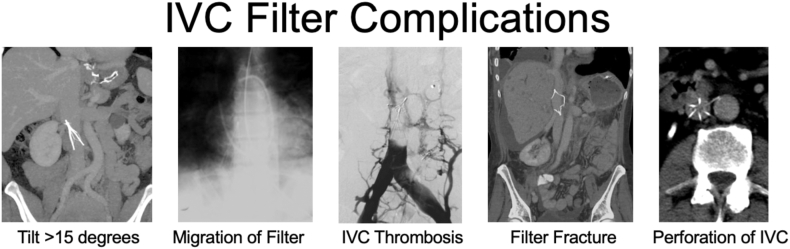
Selected complications of inferior vena cava (IVC) filters. Presented by Geoffrey Barnes, MD, at the American Society of Hematology 2023 Annual Meeting Special Session on Quality. Images adapted from Grewal et al. [10].

IVC filters are intravascular devices intended to prevent pulmonary embolism in patients at high risk for venous thromboembolism (VTE) and who are temporarily unable to tolerate anticoagulation. While IVC filters reduce the risk of pulmonary embolism, they are associated with an increased risk of deep venous thrombosis and have no impact on all-cause mortality [13]. Further, they are subject to a variety of complications, including filter migration, IVC perforation, and caval thrombosis [14] (Figure 1). The majority of filters in clinical use currently are retrievable, and early retrieval is imperative. Indwell duration greater than 30 days is an important predictor of the development of complications, and the success of retrieval attempts decreases over time [15].

Current guidelines advise prompt filter retrieval [[16], [17], [18]]. Yet, while retrieval rates have increased over time [19], recent national estimates suggest that only 17% of filters are removed [20]. Factors associated with an increased likelihood of filter removal include higher income and younger age [21]. An important barrier to filter removal is that patient education on this matter is particularly lacking. In one study of 600 patients, only 23% knew that their filter could be removed, and an additional 12% were not even aware of having a filter in place [22]. The latter finding is likely the product of IVC filters often being used in acutely ill patients with a variety of significant medical issues to manage. Thus, the impetus is on providers and/or health systems to incorporate pathways to provide better care for patients who undergo this procedure.

Multiple types of interventions to increase retrieval rates have been studied. Interventions can broadly be categorized into passive and active surveillance of IVC filter patients [23]. Examples of passive surveillance include automated nudges to patients (eg, letters, emails, phone calls, and clinic appointments) and providers (eg, indwell time alerts, follow-up flags, and reminder systems) to reconvene and pursue filter retrieval [23]. While passive surveillance has been effective in published studies and is relatively low-cost, authors of such studies have suggested that the incorporation of active surveillance is needed to meaningfully increase retrieval rates [24,25].

Active surveillance typically involves the creation of patient registries that are regularly monitored by clinical personnel. Workflows tend to involve physician supervision of nurses, advanced practice providers, or trainees who maintain the registry [[26], [27], [28], [29]]. One study observed that a transition from passive to active surveillance significantly increased retrieval rates from 49% to 61% [30]. While active intervention tends to require increased responsibilities for existing staff or hiring new staff, these programs have been shown to be cost-effective. In one report, the hospital’s cost to maintain an active surveillance program was offset by the removal of an additional 3 IVC filters per year [31].

Given the fragmented care involved in IVC filter placement and retrieval, we advocate for institutions to create systems to monitor patients who receive filters and ensure regular reassessment for removal [16,17]. While hematologists generally do not recommend IVC filters, they do frequently interact with patients who have received these devices and can be a part of institutional solutions to increase filter retrieval. Hematologists can provide leadership in creating task forces to address this issue, provide input on when filter removal is safe, and lend expert thrombosis care to these complex cases [[32], [33], [34], [35]].

## 3 Hydroxyurea Use in SCA

**Figure 2:**
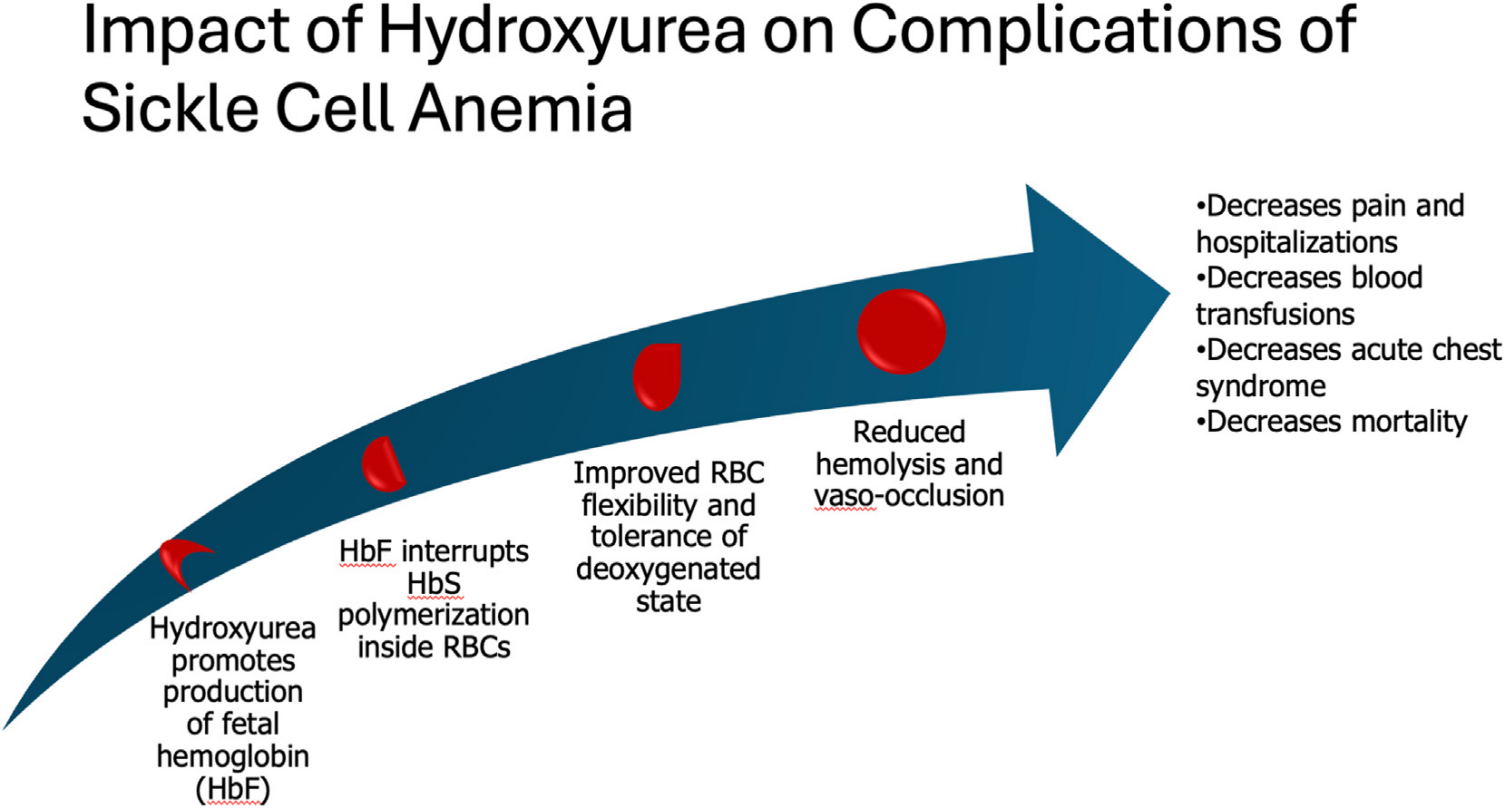
Overview of the impact of hydroxyurea on sickle cell anemia pathophysiology and complications. Presented by Alexandra Power-Hays, MD, at the American Society of Hematology 2023 Annual Meeting Special Session on Quality. Images adapted from the Centers for Disease Control and Prevention [44]. HbF, hemoglobin F; HbS, hemoglobin S; RBC, red blood cell.

Hydroxyurea is approved by the U.S. Food and Drug Administration to reduce the frequency of painful crises and blood transfusions in people with SCA (genotypes hemoglobin [Hb] SS and HbS/β-zero thalassemia) by increasing fetal hemoglobin, thereby reducing sickle hemoglobin polymerization [[36], [37], [38], [39]]. Hydroxyurea has many other benefits in people with SCA, including reduced rates of acute chest syndrome, strokes, healthcare utilization, and overall mortality [[40], [41], [42], [43]] (Figure 2), as well as being cost-effective [45]. Since 2014, the National Heart, Lung, and Blood Institute (NHLBI) has recommended that all infants more than 9 months of age, children, and adolescents with SCA be offered hydroxyurea, regardless of disease severity [46]. The NHLBI also recommends that adults with SCA and frequent or severe complications of pain, anemia, or acute chest syndrome start hydroxyurea.

Despite these recommendations, only 1 in 4 Americans with SCA who meet the NHLBI criteria for treatment are treated with hydroxyurea [42,[47], [48], [49]]. There are numerous barriers to the increased uptake of hydroxyurea. On the provider side, these include an inadequate number of clinicians knowledgeable about SCA and hydroxyurea, and bias regarding the likelihood of adherence [50]. Provider bias regarding patient adherence to hydroxyurea and monitoring has also been cited as a barrier to prescribing [50]. Addressing this will require individual introspection, department and institution-level attention, and systemic changes to support this long-neglected patient population [51,52]. On the patient side, doubts about efficacy and apprehension about long-term medication use are frequently raised [50]. Concerns about side effects are prevalent among both patients and providers. Additionally, up to 10% of patients not presently on hydroxyurea report that they tried it in the past and it did not work for them [53,54], though this may be related to insufficient dosing rather than true inefficacy or side effects, given that effective adult dosing can range as high as 35 mg/kg of body weight per day [37]. Increased hydroxyurea use in SCA requires interventions directed at both providers and patients.

Interventions targeting improved prescription rates among providers focus on the adoption of the NHLBI guidelines in institutional practice. A regional, multistate collaboration worked to promote provider adherence to the NHLBI guidelines for SCA care with a particular focus on increasing hydroxyurea prescriptions in pediatric SCA patients in the state of Florida [55]. Well-established quality improvement methodologies, such as plan-do-study-act cycles, were utilized at multiple clinics, tailored to the needs of each specific center. Providers received coaching to overcome barriers to prescribing and emphasized the need to deliver ongoing patient education. The intervention resulted in an increased proportion of patients with a hydroxyurea prescription (50%-59%) across multiple clinical settings. A single-institution study achieved near-universal hydroxyurea use (43%-95%) among pediatric patients with SCA by updating institutional guidelines to match the NHLBI guidelines, conversations about benefits and risks starting in infancy, and holistic support of patients and families by ensuring clinic staff included knowledgeable clinicians, social workers, nurse care managers, school support liaisons, and psychologists. Coinciding with the increase in hydroxyurea treatment, there was nearly a 50% reduction in SCA-related hospital admissions [56].

To facilitate patient-provider communication about hydroxyurea, Smith et al. [57] implemented a dedicated hydroxyurea informed consent procedure in the clinic workflow. The consent process was designed to address the specific concerns shared by many patients in the predominantly Black SCA community, including hair loss, oncologic risk, pregnancy risk, and the necessity of taking a daily medication even when not experiencing symptoms. The revised consent procedure emphasized verbal rather than written communication, and language was modified to avoid overly complex medical terminology. Initially, 17 of 96 eligible clinic patients (18%) were on hydroxyurea. After the intervention, this increased to 44 of 96 patients (46%).

To attempt to ensure appropriate intensity of hydroxyurea dosing, Roessner et al. [58] implemented a pharmacist-led protocol to support the uptake of hydroxyurea among eligible patients and optimize dosing. Patients started on hydroxyurea were monitored by pharmacists, who adjusted doses as appropriate and counseled patients regularly. As a result, the proportion of patients on the maximum tolerated dose of hydroxyurea increased from 35% to 64%. On average, 3 dose increases and 8 pharmacist interventions were needed to reach the maximum tolerated dose, underscoring the need for focused longitudinal attention to this issue.

In attempting to overcome patient-level barriers, multiple studies have utilized mobile health technologies designed to promote hydroxyurea adherence. Automated text message reminders were shown to result in improvements in hematologic parameters [59]. A smartphone application capable of providing daily customizable reminders, reporting hydroxyurea adherence to providers, and linking inpatient accountability partners demonstrated an increase in hydroxyurea adherence from 40% to 56% [60]. A program for directly observed therapy involving text message reminders and daily video reporting of medication administration similarly increased adherence from 30% to 67% [61]. Alert fatigue was cited as a barrier to the success of these types of interventions. Of note, one study also evaluated a mobile health intervention targeted at providers, which proved ineffective [60]. The authors postulated that providers viewed themselves as SCA experts and did not expect to derive benefits from using mobile health tools.

While there are many exciting developments in the SCA world, such as gene therapy, the low uptake of hydroxyurea is a missed opportunity to provide effective and inexpensive therapy accessible to all patients, particularly given the lack of barriers to hydroxyurea use present in other parts of the world (eg, equipment for laboratory monitoring) [62]. The interventions described above are examples of ways to overcome barriers to higher uptake specific to providers and patients. It is clear that without dedicated efforts, hydroxyurea utilization will remain suboptimal, and outcomes for patients with SCA will suffer.

## 4 Patient Education Prior to Anticoagulation

Oral anticoagulants (OACs) are used in the treatment and prevention of VTE and the prevention of stroke in patients with atrial fibrillation. OACs are classified as high-risk medications due to the high potential for harm if used improperly. In 2019, the Joint Commission implemented 8 new elements of performance related to OAC use as part of their National Patient Safety Goals to reverse the rising rates of OAC-related adverse events [63]. One of the elements includes providing education to patients and families specific to the OAC prescribed. There is limited direct evidence that patient education improves patient outcomes. Most are single-institution studies that have found a correlation between targeted in-person education and patients’ knowledge level, and a correlation between patients’ knowledge of OACs and medication adherence [[64], [65], [66], [67]]. One randomized controlled study of 152 participants found that pharmacist-led education led to better OAC knowledge and anticoagulation control (time within the target range for warfarin) [68]. As such, the American Society of Hematology guidelines for management of VTE included a recommendation that *suggests* using supplementary patient education in addition to basic education (conditional recommendation based on very low certainty in the evidence about effects) for patients receiving OACs [69].

Yet, despite these requirements and recommendations, patient education is often underused. An ambulatory cancer clinic found that fewer than 5% of patients with cancer initiating any cancer-directed therapy received any VTE education (VTE risk and signs and symptoms of thrombosis) [70]. On a global level, a survey of 745 cancer patients found that only one-third of respondents had received any VTE education [71]. There are perceived barriers to offering patient education, including physician time famine, resource constraints, and workflow deviations.

There is significant heterogeneity in the type of patient education provided that varies in modality, content, and intensity [72]. A survey of current anticoagulation patient education practices found that all providers used printed supplemental education materials, whereas videos and computer-based modules were used by 14.8% and 8.2%, respectively [73]. One study found that the use of a standardized patient education video, which was viewed while patients were in the waiting room, significantly reduced the time spent on face-to-face counseling at an outpatient anticoagulation clinic by up to 25% without impacting patient satisfaction [74]. As such, increased use of educational videos may help overcome the barrier of time constraints in providing patient education.

Although patient education prior to initiating anticoagulation is usually provided by the physician, the impact of pharmacists in providing patient education cannot be understated. In one study of patients discharged from the emergency department (ED) with a new prescription for any OAC, patients who received ED pharmacist education were less likely to be readmitted to the hospital or return to the ED within 90 days after their initial visit for an anticoagulation-related problem (1.85% vs 12.12%; *P* = .0069) compared with those who only received physician or nursing-driven discharge instructions [75]. This pharmacist discharge education program was relatively straightforward and easily implemented within the ED’s discharge process without causing any workflow deviations.

**Figure 3:**
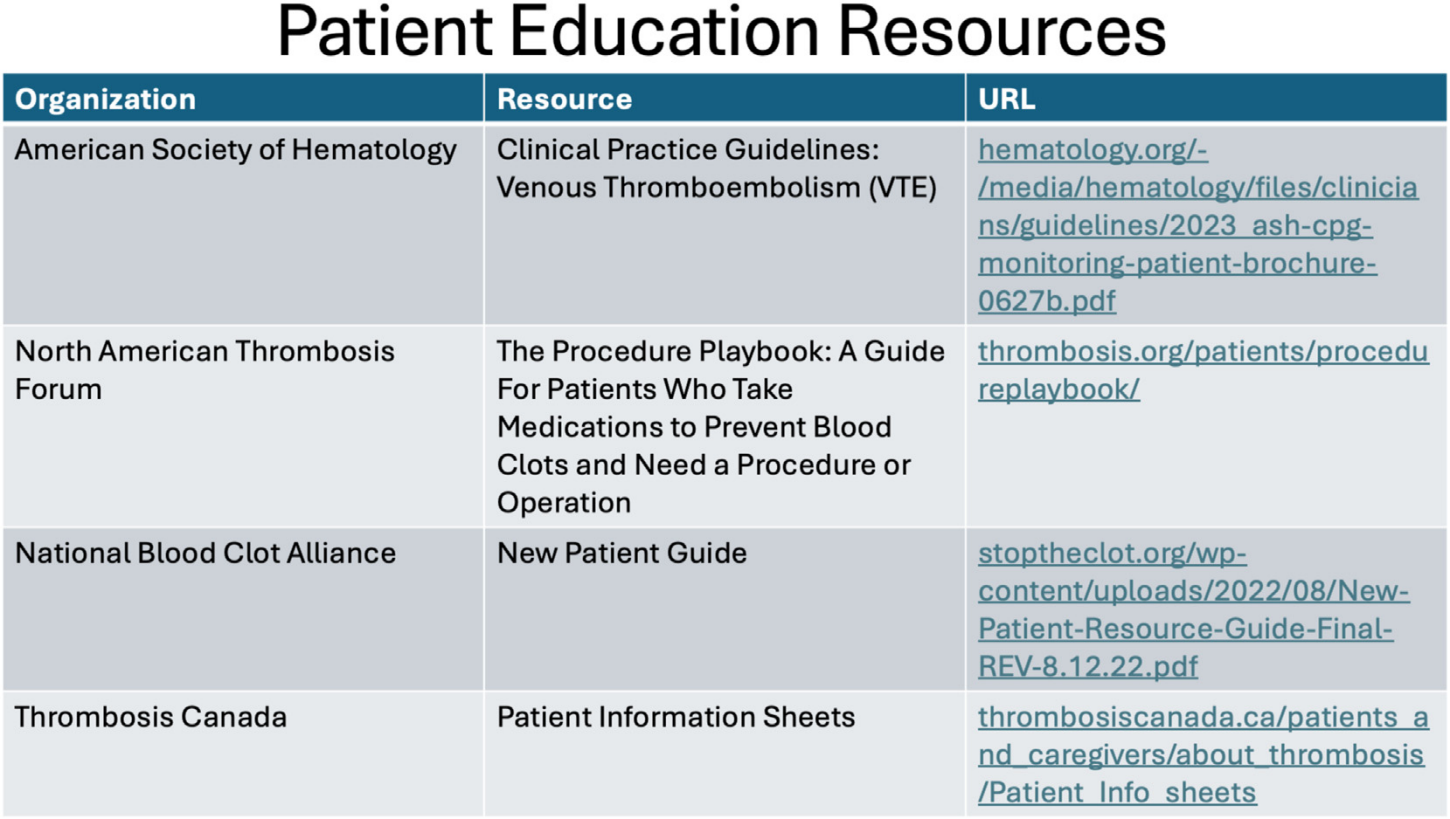
Resources for patient education prior to anticoagulation. Presented by Allison Burnett, PharmD, PhC, CACP, at the American Society of Hematology 2023 Annual Meeting Special Session on Quality. VTE, venous thromboembolism.

Given the variability in clinical practices, it is not possible to recommend one type of patient education that would be applicable to all. Similarly, it is not possible for any specialty to be solely tasked with providing patient education, given that anticoagulation is prescribed by various providers, including ED physicians, primary care physicians, hospitalists, hematologists, cardiologists, and pulmonologists. Instead, we advocate for enhancing providers’ awareness of education resources and practices locally. There are multiple resources on OAC use available online, which can be adapted for local use (Figure 3). We also advocate for providers to contribute to the development of educational tools that address not only the OACs but also social determinants of health that impact optimal anticoagulation care [76]. Jones et al. [77] developed a complementary encounter and patient decision aid for anticoagulation choice and stroke prevention in atrial fibrillation. This decision aid focused on personalized stroke risk, differences between anticoagulants, and risks of bleeding. A clinical trial on the efficacy of this decision aid is currently ongoing [77].

More importantly, we advocate for providers to act as champions within their institution for the prioritization of standardized, system-level patient education processes. With increased institutional investment in anticoagulation stewardship programs, supported by national organizations such as the Anticoagulation Forum (https://acforum.org/), the National Blood Clot Alliance (https://www.stoptheclot.org/), and the Venous Thromboembolism Network U.S. (https://venusresearch.org/), there is an opportunity to embed educational efforts within such programs for dissemination and implementation. With increased institutional investment in anticoagulation stewardship programs, supported by national organizations such as the Anticoagulation Forum, there is an opportunity to embed educational efforts within such programs. The University of New Mexico, for instance, developed a standardized, systems-based process for anticoagulation patient education practices (unpublished data). Using a form embedded within their electronic medical records, both nurses and pharmacists document how the education was given (explanation, demonstration, handout, video, etc.). Through this process, they have been able to track process measures, such as the proportion of patients with documented discharge anticoagulation education, provide feedback to staff on their performance, and improve via a plan-do-study-act approach.

## 5 Conclusion

In the North American hematology community, the overuse of unnecessary medical services has received comparatively more attention than the underuse of effective care. In this review, we have summarized published literature regarding underutilized hematologic interventions in procedural, medical, and educational domains. Our hope is that this review can guide institutions in efforts to increase the uptake of these and other forms of underutilized care.

Efforts to enhance the provision of underused care can be difficult, as they can require financial investments by institutions. By comparison, reducing overused care is often more appealing, as these efforts can promote cost savings. However, coordinated efforts to remove IVC filters, increase hydroxyurea use, and educate patients starting on anticoagulation can prevent future patient harm and associated costs. We advocate for enlisting systems-based hematologists to devise interventions to promote such care.

National societies can aid these efforts as well. With this article as a starting point, we suggest the development of a Choosing Wisely list of underused hematologic services. This list could serve as a useful companion to the list of overused services currently in existence [3,4]. The past decade of experience with attempts to the implementation of the initial overuse-focused list could serve to guide future efforts to increase underused practices that are known to have benefits, which justify any associated risks or financial costs [9].
